# Fabrication of Completely Polymer-Based Solar Cells with p- and n-Type Semiconducting Block Copolymers with Electrically Inert Polystyrene

**DOI:** 10.3390/ma11030343

**Published:** 2018-02-27

**Authors:** Eri Tomita, Shinji Kanehashi, Kenji Ogino

**Affiliations:** Graduate School of Bio-Applications Systems Engineering, Tokyo University of Agriculture and Technology, 2-24-16 Nakacho, Koganei, Tokyo 184-8588, Japan; s166994x@st.go.tuat.ac.jp (E.T.); kanehasi@cc.tuat.ac.jp (S.K.)

**Keywords:** organic photovoltaic, all-polymer solar cell, block copolymer, poly(3-hexylthiophene) (P3HT), P(NDI2OD-T2), polystyrene, semiconducting polymer, domain size, charge mobility

## Abstract

It is widely recognized that fullerene derivatives show several advantages as n-type materials in photovoltaic applications. However, conventional [6,6]-phenyl-C61-butyric acid methyl ester (PCBM) exhibits weak absorption in the visible region, and poor morphological stability, due to the facile aggregation. For further improvement of the device performance and durability, utilization of n-type polymeric materials instead of PCBM is considered to be a good way to solve the problems. In this study, we fabricated completely polymer-based solar cells utilizing p- and n-type block copolymers consisting of poly(3-hexylthiophene) (P3HT) and poly{[*N*,*N′*-bis(2-octyldodecyl)naphthalene-1,4,5,8-*bis*(dicarboximide)-2,6-diyl]-*alt*-5,5′-(2,2′-bithiophene)} [P(NDI2OD-T2)], respectively, containing common polystyrene (PSt) inert blocks, which decreased the size of phase separated structures. Electron mobility in synthesized P(NDI2OD-T2)-*b*-PSt film enhanced by a factor of 8 compared with homopolymer. The root mean square roughness of the blend film of two block copolymers (12.2 nm) was decreased, compared with that of the simple homopolymers blend (18.8 nm). From the current density-voltage characteristics, it was confirmed that the introduction of PSt into both P3HT and P(NDI2OD-T2) improves short-circuit current density (1.16 to 1.73 mA cm^−2^) and power-conversion efficiency (0.24% to 0.32%). Better performance is probably due to the uniformity of the phase separation, and the enhancement of charge mobility.

## 1. Introduction

**Figure 1:**
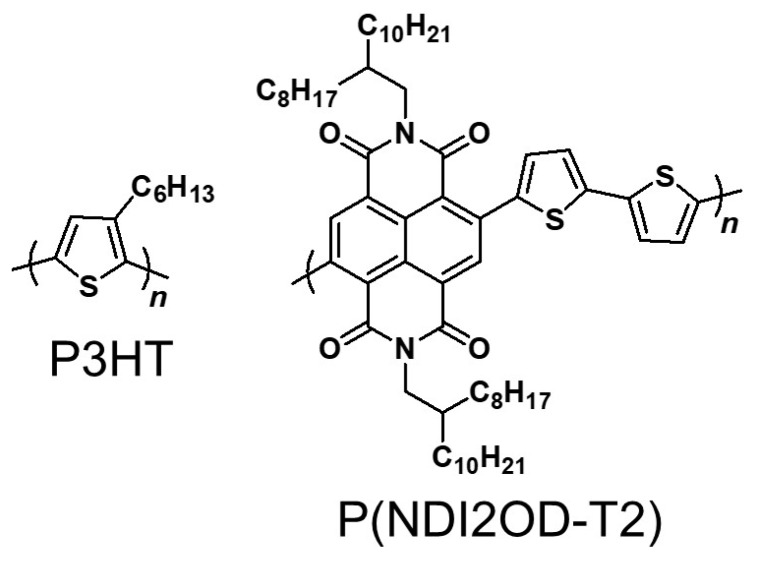
Chemical structures of P3HT and P(NDI2OD-T2).

Organic photovoltaics (OPVs) have attracted much attention as a new clean energy source, since the devices possess light, and flexible natures, and they can be fabricated at low-cost in large areas suitable for mass-production [1]. In general, the active layer of a polymer-based OPV is considered to possess a bulk-heterojunction (BHJ) structure in which electron donor (p-type material) and acceptor (n-type one) are interpenetrated each other [2]. OPVs can extract electrical energy from light via following four steps, (1) absorption of light and generation of excitons, (2) diffusion of the excitons to the p-n interfaces, (3) dissociation of the excitons to generate free charges, a hole-electron pair, (4) charge transporting, and collection of hole and electron by each electrode [3]. Most OPVs studied so far have utilized low molecular weight fullerene derivatives as n-type materials [4,5]. Since fullerene derivatives have several disadvantages such as high cost, low light absorption in the visible region, and low compatibility, studies on all-polymer solar cells utilizing polymeric acceptors, rather than low molecular weight fullerene derivatives, have been advanced in recent years [6,7,8,9,10,11,12,13,14,15]. In the case of non-fullerene polymeric alternatives, molecular versatility makes it possible to design suitable n-type polymeric semiconductors that exhibit high light absorption in the visible region in order to harvest a large portion of sunlight, resulting in a high short-circuit current density (*J*_SC_) [9]. Furthermore, by adjusting the energy levels of the highest occupied molecular orbital (HOMO) and the lowest unoccupied molecular orbital (LUMO), it is possible to fulfil a high open-circuit voltage (*V*_OC_) [9]. Among the various polymeric acceptors used in completely polymer-based BHJ solar cells, poly{[*N*,*N′*-*bis*(2-octyldodecyl)-naphthalene-1,4,5,8-bis(dicarboximide)-2,6-diyl]-*alt*-5,5′-(2,2′-bithiophene)} [P(NDI2OD-T2)] (Figure 1) has been recognized as one of the most promising materials, having naphthalene diimide moieties which are known to have high absorption in the visible region, and high electron affinity with appropriate energy level [16]. It was reported that the BHJ completely polymer-based solar cells having active layers using the combination of P(NDI2OD-T2) with the most common p-type poly(3-hexylthiophene) (P3HT) (Figure 1) prepared from dichlorobenzene solution (as-spun) showed a power-conversion efficiency (PCE) of 0.17% [10].

There are several strategies to improve the PCE, such as increasing the amount of light absorption in the visible region and the charge separation efficiency, and the enhancement of the charge mobility [3]. In order to increase the charge separation efficiency, it is effective to increase the heterojunction interface between p- and n-domains [11]. The larger interface can be achieved by reducing the domain size of the phase separation of p- and n-type polymers [12]. Since different polymers are generally incompatible, the blending affords phase separated films with various domain sizes dependent on the fabrication conditions. As a conventional and easy method, the optimization of the solvent used in the fabrication of the active layer has been carried out [17]. For example, by changing the solvent from chlorobenzene with its higher boiling point to more volatile chloroform in the fabrication of P3HT/P(NDI2OD-T2) blend film, the domain size was decreased and a high *J*_SC_ was achieved, resulting in high PCE [10]. As a similar example, in the OPV system in which P3HT and poly{2,7-(9,9-didodecylfluorene)-*alt*-5,5-[4′,7′-bis(2-thienyl)-2′,1′,3′-benzothiadiazole]} are used as p- and n-type polymers, respectively, the film morphology changes with the spin-coating solvent. The size of the phase separation decreases in the order of dichlorobenzene, chlorobenzene, and chloroform. Contrary to the conventional devices based on a p-type polymer and [6,6]-phenyl-C61-butyric acid methyl ester (PCBM), the thermal annealing provides the slight changes of the interfacial morphology due to the lack of enough mobility in the case of polymer blended system [13].

While the morphological control by the choice of the solvent relies on kinetics of phase separation or the rate of the solvent evaporation, a thermodynamically controlled method for reducing the domain size is a promising alternative. Our strategy here is based on the idea of introducing a common or similar structure to each component to reduce the domain size. In the case of binary blends consisting of asymmetrical AB and AC type block copolymers, where the composition of common A segments are lower than B or C, the macrophase separation affording B- and C-rich domains is expected to proceed accompanied with the microphase separation in each macro domain [18]. Simultaneously it is anticipated that the part of micophase separated A domain exists at the phase boundary between the macrodomains to reduce the interfacial energy making the domain size small. 

In this paper, in order to improve the uniformity of the phase separation, and obtain a high PCE, we attempted to increase the affinity between p- and n-type polymers by introducing a common block to both p- and n-type polymers based on the above-mentioned strategy. As p- and n-type polymers, conventional P3HT and P(NDI2OD-T2) with high light absorption ability in the visible region, and relatively high charge mobility were utilized, respectively. As a common block, electrically inert polystyrene (PSt) was used. There is a possibility that the introduction of PSt alters the electric characteristics as well as the domain size. We recently reported that the hole mobility of P3HT is improved by the introduction of PSt [19]. Therefore, it is expected that the enhancement of charge mobilities can also contribute to the improvement of PCE.

## 2. Materials and Methods

### 2.1. Materials

#### 2.1.1. P3HT-*b*-PSt

The p-type semiconducting P3HT bearing a bromo terminal group at one end was synthesized by Grignard-metathesis reaction according to the literature [20]. The number average molecular weight *M*_n_ and polydispersity index (PDI) of P3HT were 12,000 g mol^−1^ and 1.73, respectively. The boronic ester terminated PSt was synthesized by atom transfer radical polymerization (ATRP) as previously reported [21]. The *M*_n_ and PDI of PSt for P3HT-*b*-PSt synthesis were 2220 g mol^−1^ and 1.25, respectively. The block copolymer, P3HT-*b*-PSt was synthesized by Suzuki-Miyaura coupling of bromo terminated P3HT with boronic ester terminated PSt as previously reported [22]. The *M*_n_ and PDI of P3HT-*b*-PSt were 13,100 g mol^−1^ and 1.90, respectively. The weight ratio of PSt segments in P3HT-*b*-PSt was estimated to 11.0%. A similar block copolymer containing low molecular weight of P3HT-l (*M*_n_ = 4230 g mol^−1^, PDI = 1.59), and the same PSt segments (P3HT-l-*b*-PSt) was also synthesized. The weight ratio of the PSt segments in P3HT-l-*b*-PSt was estimated to be 28.0%.

#### 2.1.2. P(NDI2OD-T2)-Br_2_

**Figure 2:**
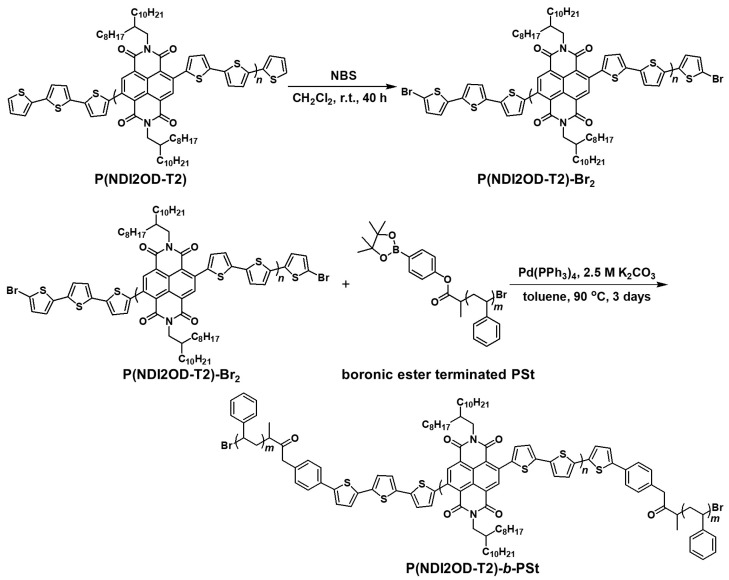
Synthetic scheme of P(NDI2OD-T2)-*b*-PSt.

An n-type semiconducting P(NDI2OD-T2) was synthesized by Stille coupling reaction with a modified method in the literature (see Figure S1 in Supplementary Materials) [23,24,25,26,27]. In order to ensure the terthiophene chain ends as shown in Figure 2, the subsequent termination reaction with 2-bromothiophene was conducted. The *M*_n_ and PDI of P(NDI2OD-T2) were 10,100 g mol^−1^ and 3.33, respectively. P(NDI2OD-T2)-Br_2_ was synthesized as follows. P(NDI2OD-T2) (380 mg, 0.038 mmol) and CH_2_Cl_2_ (6 mL) were placed into a 50-mL of flask. *N*-bromosuccinimide (14.8 mg, 0.083 mmol) was added, then the mixture was stirred for 40 h at room temperature. The product was precipitated in acetone. Yield: 372 mg (98%).

#### 2.1.3. P(NDI2OD-T2)-*b*-PSt

The boronic ester terminated PSt with different molecular weight was synthesized by ATRP in a similar manner as the above mentioned. The *M*_n_ and PDI of PSt for P(NDI2OD-T2)-*b*-PSt preparation were 620 g mol^−1^ and 1.94, respectively. The block copolymer, P(NDI2OD-T2)-*b*-PSt was synthesized by Suzuki-Miyaura coupling of P(NDI2OD-T2)-Br_2_ with boronic ester terminated PSt as shown in Figure 2. The detail of synthesis was as follows.

P(NDI2OD-T2)-Br_2_ (206 mg), boronic ester terminated PSt (49 mg), Pd(PPh_3_)_4_ (23 mg, 0.020 mmol), 2.5 M K_2_CO_3_ (2 mL), and toluene (10 mL) were placed into a 50-mL of two necked flask equipped with a condenser and a nitrogen inlet, followed by freeze-and-thaw cycles to eliminate air in the mixture. Then the mixture was stirred for 3 days at 90 °C under nitrogen atmosphere. The reaction mixture was precipitated in methanol to recover the product. Reprecipitations in methanol and hot acetone were successively carried out twice each. Then, Soxhlet extraction was carried out using in the order of methanol, acetone and chloroform, and a solution of chloroform was poured into methanol to recover the final product. Yield: 224 mg (90%). The *M*_n_ and PDI of P(NDI2OD-T2)-*b*-PSt were 12,200 g mol^−1^ and 5.59, respectively. The weight ratio of PSt segments in P(NDI2OD-T2)-*b*-PSt was 5.9%. Similar block copolymer containing low molecular weight of P(NDI2OD-T2)-l (*M*_n_ = 3880 g mol^−1^, PDI = 1.94), and the same PSt segments (P(NDI2OD-T2)-l-*b*-PSt) was also synthesized as mentioned above. The weight ratio of PSt segments in P(NDI2OD-T2)-l-*b*-PSt was 26.9% (see Figure S2 in Supplementary Materials).

### 2.2. Characterizations

Syntheses of all compounds were confirmed by NMR spectroscopy (JEOL, JNM-ECX300 or JEOL JNM-ECA500, Tokyo, Japan). The values of *M*_n_ and PDI were determined by gel permeation chromatography (GPC) using a system equipped with a set of two columns (7.6 mm Φ × 30 cm) packed with home-made styrene-divinylbenzene gel beads [28] and a UV detector (254 nm) (JASCO, UV-2075 Plus, Tokyo, Japan) using chloroform as an eluent (0.5 mL/min), which was calibrated against monodispersed polystyrene standards. The values of weight ratio of PSt were determined from ^1^H NMR spectrum. 

The measurements of cyclic voltammetry (CV) for film samples were carried out on a potentiostat/galvanostat (HOKUTO DENKO, HZ-5000, Tokyo, Japan) at a scan rate of 100 mV s^−1^, where acetonitrile and 0.1 M tetrabutylammonium perchlorate were used as a solvent and electrolyte, respectively. Platinum wires and carbon were used as a counter and working electrodes, respectively. Ag/AgCl was utilized as a reference electrode. The thin films were prepared by the drop casting of the sample solutions with a concentration of 20 mg mL^−1^ on the working electrode. The UV-vis absorption spectra were obtained with a JASCO V-670 spectrophotometer (Tokyo, Japan). Thin films of blends, P3HT/P(NDI2OD-T2) and P3HT-*b*-PSt/P(NDI2OD-T2)-*b*-PSt, were prepared by spin coating from chlorobenzene solution on glass substrates on the same conditions as those of photovoltaic devices. Thin films of P3HT and P(NDI2OD-T2) were prepared by spin coating of chlorobenzene solution of which the concentration was maintained at 13 mg mL^−1^ and 12 mg mL^−1^, respectively, to keep the absorption intensity comparable to those of photovoltaic devices.

The nanoscale morphology of the blended films of the devices was investigated using atomic force microscopy (AFM) (SII, Nanocute, Tokyo, Japan) using silicon probes with a resonant frequency of ≈300 kHz and a force constant of ≈26 N m^−1^ (Olympus, OMCL-AC160TS, Tokyo, Japan). The crystal structure of the blended films on the glass was measured by grazing incidence wide angle X-ray diffraction (GIWAXD) (RIGAKU X-ray Diffractometer SmartLab, Tokyo, Japan) (Cu Kα, λ = 1.5418 Å, 45 kV and 200 mA) from 2° to 30° with a step of 0.02° at the scan speed of 1° min^−1^ in the out-of-plane measurements. Incident angle was fixed to 0.20°. The thickness of the films and metals was measured using a Stylus type surface profiler (BRUKER, Dektak XT-S, Billerica, MA, USA).

### 2.3. Device Fabrication of Space Charge Limited Current (SCLC) Measurements

The electron only devices were fabricated with a configuration of indium tin oxide (ITO)/aluminum (Al) (50 nm)/active layer (200 nm)/lithium fluoride (LiF) (0.5 nm)/Al (100 nm). ITO-coated glass substrates (10 Ω per square) were washed by an alkaline cleaner under sonication and rinsed with deionized water. Al with 50 nm of thickness was vacuum-deposited at 3.0 × 10^−4^ Pa onto the ITO substrate. Active layers were spin-coated from chlorobenzene solutions having a concentration of 40 mg mL^−1^ that were filtered with a 0.45 μm membrane filter. The thickness of the polymer layers was typically adjusted to 200 nm. Then, LiF with 0.5 nm of thickness and Al with 100 nm of thickness were successively vacuum-deposited at 3.0 × 10^−4^ Pa. A typical size of the photo-active area was 6 mm^2^. The current-voltage characteristics of the devices were measured by using a direct-current voltage and a current source/monitor (KEITHLEY, 2400, Cleveland, OH, USA).

### 2.4. Fabrication of OPV (J-V) Device and Measurements

All the devices were fabricated with a structure of ITO/poly(3,4-ethylenedioxythiophene) (PEDOT):poly(4–styrenesulfonate) (PSS) (30 nm)/active layer (150 nm)/LiF (0.5 nm)/Al (100 nm). ITO-coated glass substrates (10 Ω per square) were cleaned in the same way as the SCLC devices. The substrate was subsequently washed by 2-propanol under sonication, rinsed with clean 2-propanol, and dried with nitrogen. PEDOT:PSS (Heraeus, Clevious P VP Al 4083, Hanau, Germany) was spin-coated onto the ITO substrate at a spinning rate of 2500 rpm (rotations per min) for 60 s from the dispersion in water filtered by a 0.2 μm of membrane filter, followed by dried in air at 200 °C for 1 h. All polymer blended layers were spin-coated from chlorobenzene solutions filtrated with a 0.45 μm of membrane filter. The total concentration of these blended solutions, in which p- and n-type polymers were dissolved at weight ratios 13:12, was maintained at 25 mg mL^−1^ to keep the film thickness constant. The thickness of the polymer blended layers was typically 150 nm. Then, LiF with 0.5 nm of thickness and Al with 100 nm of thickness were vacuum-deposited at 3.0 × 10^−4^ Pa. A typical size of the photo-active area was 5.6 mm^2^. The photocurrent-voltage characteristics of the devices were measured by using a direct-current voltage and a current source/monitor (KEITHLEY, 2400, Cleveland, OH, USA) under illumination by air mass 1.5G-Grobal (AM1.5G) simulated solar light by a xenon lump with a power of 100 mW cm^−2^.

## 3. Results and Discussion

### 3.1. Synthesis of P(NDI2OD-T2)-b-PSt 

**Figure 3:**
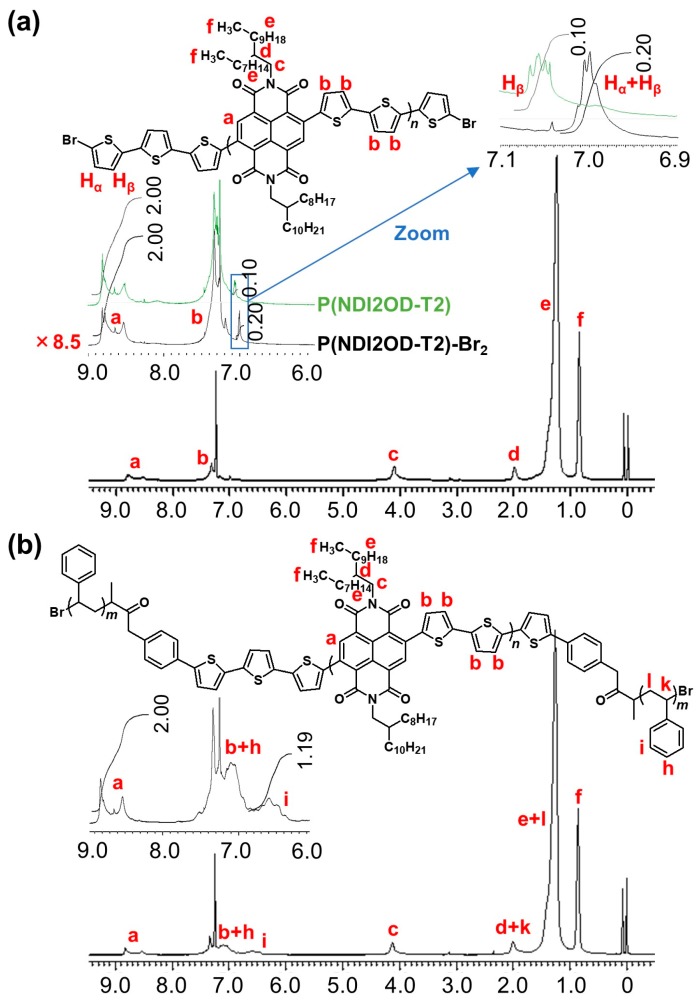
^1^H NMR spectra of (**a**) P(NDI2OD-T2)-Br_2_, (**b**) P(NDI2OD-T2)-*b*-PSt in CDCl_3_ at 500 MHz. Red letters designate the signal assignments shown in chemical structure of each polymer.

The Stille coupling reaction employed for synthesis of P(NDI2OD-T2) was terminated with 2-bromothiophene in order to obtain well-defined end groups for the preparation of the block copolymer. Figure 3a shows the ^1^H NMR spectrum of P(NDI2OD-T2)-Br_2_, and the spectrum pattern was similar to the original P(NDI2OD-T2) except the signal from chain ends. In the ^1^H NMR spectrum of P(NDI2OD-T2) (see Figure S1 in Supplementary Materials), the signals for β-hydrogen from end thiophene units as designated in Figure 3a were separately observed around 7.05 ppm (inset of Figure 3a), and the signal assignment is based on the spectrum for tetrathiophene [29]. After bromination, the signals derived from the chain ends were observed at the slightly higher magnetic field compared with the original polymer, and the relative intensity increased by a factor of 2. These observations were also reasonably explained on the basis of the signal assignments for brominated tetrathiophene [29], where the signals both of α- and β-hydrogens show higher magnetic field shifts from 7.22 to 6.98 ppm, and from 7.02 to 6.91 ppm respectively. Assuming that these shifts are similarly true for P(NDI2OD-T2)-Br_2_, it is considered that the signals around 7.0 ppm are derived from the overlap of the signals of α- and β-hydrogens. Consequently, from these NMR analyses, it is considered that bromination at end groups occurred quantitatively. Furthermore, the degree of polymerization can be estimated to 20 from the comparison of the intensity of signal “**a**” from naphthalene diimide unit to that of the end thiophene.

As shown in Figure 3b, additional signals in the aromatic region assignable to the protons on the phenyl ring of PSt are observed at 6.6 and 7.1 ppm in the ^1^H NMR spectrum of P(NDI2OD-T2)-*b*-PSt. As is the case in other block copolymers, spectrum is inherently the same as the superimposed spectra of each homopolymer. The ratio of unit numbers (corresponding to the chemical composition) was determined to 20/12, which can be converted to the weight ratio of 19,800/1240 based on the mass number of each unit. The *M*_n_ of used PSt is 620 g mol^−1^ as mentioned in the Section 2.1. Since the block copolymer possesses PSt chains at both ends, the weight ratio of 19,800/1240 is estimated, and this ratio is consistent with one determined from the chemical composition. 

### 3.2. Characteristics of P(NDI2OD-T2)-b-PSt

The electrochemical characteristics of P(NDI2OD-T2)-*b*-PSt were investigated by cyclic voltammetry. The energy levels of LUMO of homo- and block copolymers were estimated to be −4.0 and −4.1 eV (Table 1), respectively, from the onset of reduction waves in the cyclic voltammograms (see Figure S3 in Supplementary Materials). LUMO level is almost the same as the reported value (−4.0 eV) [30], and the introduction of PSt has a negligible effect on the electrochemical nature as expected, since the PSt segment is electrically inert. Wavelengths of absorption maxima in the solution state determined from UV/vis absorption spectra are also listed on Table 1, indicating that there is no significant effect of the PSt segment on the electron configuration in the ground state. 

**Figure 4:**
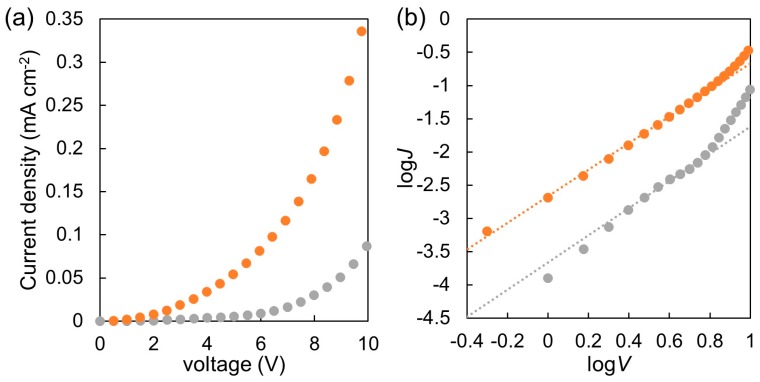
Current density-voltage (**a**) and double logarithmic plots (**b**) for electron only devices based on P(NDI2OD-T2) (gray) and P(NDI2OD-T2)-*b*-PSt (orange). The presented data set affords the electron mobilities close to the average values.

Figure 4 shows the original (a) and double logarithmic (b) plots for current density-voltage characteristics of the films of P(NDI2OD-T2) and P(NDI2OD-T2)-*b*-PSt. Electron mobility was determined with the following Equation (1) taking the series resistance (12 Ω) into consideration, and assuming built-in voltage is close to zero due to the small difference of work functions for both electrodes [31].

(1) $$J=\frac{9}{8}\varepsilon_{r}\varepsilon_{0}\mu_{e}\frac{V^{2}}{L^{3}}$$

where, *J* is the electron current density, *μ*_e_ the electron mobility, *ε*_r_ the relative permittivity of the material (3.5), *ε*_0_ the permittivity of vacuum, *L* the thickness of the active layer, and *V* voltage drop across the device. The electron mobilities determined with the Equation (1) are listed in Table 1. Compared with the P(NDI2OD-T2) precursor, it is found that P(ND2OD-T2)-*b*-PSt exhibits higher electron mobility by a factor of 8. We also reported that the introduction of the PSt block to P3HT enhances the hole mobility by a factor of over 100 [19]. In the case of P3HT-*b*-PSt, the content of the rigid amorphous domain is higher than the P3HT precursor, and this type of domains interconnect the crystallites, leading to enhancement of hole mobility. Hitherto, it is not clear that this consideration is applicable to the P(ND2OD-T2) family. Further investigation is necessary to elucidate the origin of enhanced electron mobility. 

### 3.4. UV/vis Absorption in Film State

**Figure 5:**
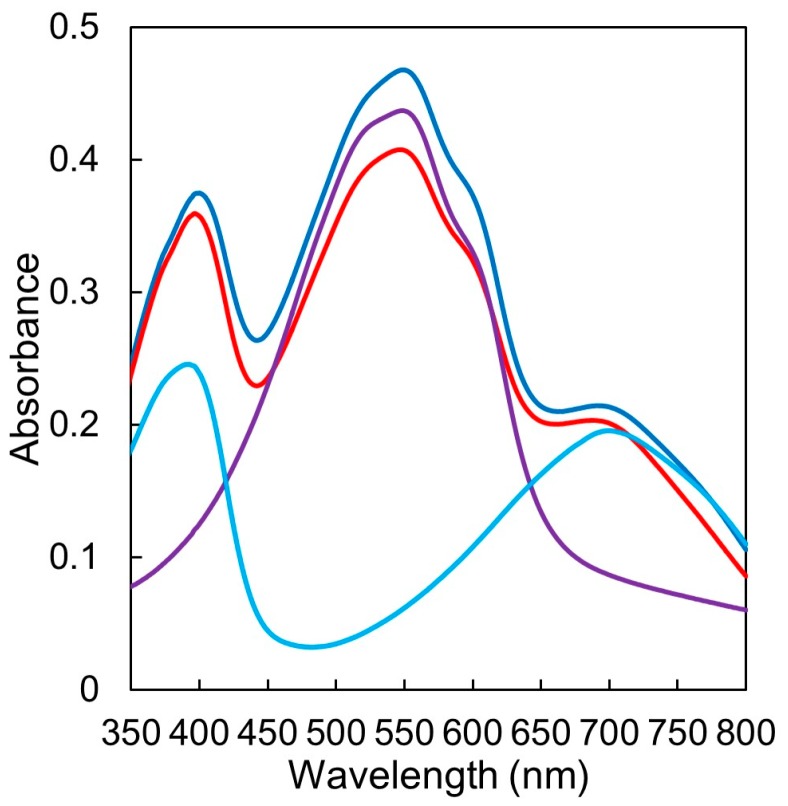
UV/vis absorption spectra of P3HT/P(NDI2OD-T2) (blue), P3HT-*b*-PSt/P(NDI2OD-T2)-*b*-PSt (red), P3HT (purple), P(NDI2OD-T2) (light blue) in films.

Figure 5 shows UV/vis absorption spectra of as-spun films of P3HT/P(NDI2OD-T2) (150 nm), P3HT-*b*-PSt/P(NDI2OD-T2)-*b*-PSt (150 nm), P3HT (80 nm) and P(NDI2OD-T2) (80 nm). Both spectra of P3HT/P(NDI2OD-T2) and P3HT-*b*-PSt/P(NDI2OD-T2)-*b*-PSt are superimposed spectra of P3HT and P(NDI2OD-T2) inherently. Comparing the spectra of P3HT/P(NDI2OD-T2) and P3HT-*b*-PSt/P(NDI2OD-T2)-*b*-PSt, the intensity of the latter is smaller than that of P3HT/P(NDI2OD-T2), which is considered to be attributable to the presence of PSt. Actually, it can be seen that for the spectrum of P3HT-*b*-PSt/P(NDI2OD-T2)-*b*-PSt compared with that of P3HT/P(NDI2OD-T2), the peaks derived from the P3HT segment with a high content of PSt greatly decreases the intensities compared with those derived from P(NDI2OD-T2) with a low content of PSt. No change in peak positions and the profiles by the introduction of PSt and blending p- and n-type of polymers was observed, and it is considered that there is no charge transfer interaction at least in the ground state in the blend films.

### 3.5. Morphology

**Figure 6:**
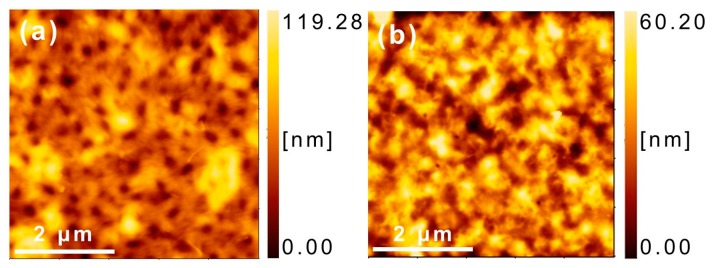
Atomic force microscopy (AFM) height images (5 μm × 5 μm) of (**a**) P3HT/P(NDI2OD-T2) and (**b**) P3HT-*b*-PSt/P(NDI2OD-T2)-*b*-PSt.

The AFM height images are shown in Figure 6. It is found that the film of P3HT/P(NDI2OD-T2) exhibits a relatively rougher surface with the root mean square (RMS) roughness of 18.8 nm. On the other hand, the film of P3HT-*b*-PSt/P(NDI2OD-T2)-*b*-PSt exhibits a RMS of 12.2 nm. Thus, the value of RMS the film of the blend of p- and n-type polymers, in which the common PSt block segment was introduced, decreased. From the RMS data, it was shown that P3HT-*b*-PSt/P(NDI2OD-T2)-*b*-PSt afford smoother film surface than P3HT/P(NDI2OD-T2), which means that more uniform films without large aggregations can be formed [32]. Furthermore, judging from the images of both polymers, P3HT-*b*-PSt/P(NDI2OD-T2)-*b*-PSt film gives relatively uniform phase separation, while in P3HT/P(NDI2OD-T2) film, there exist some bigger aggregations. This is probably due to the improvement of compatibility by the existence of PSt segment in both polymers P3HT-*b*-PSt and P(NDI2OD-T2)-*b*-PSt.

### 3.6. Photovoltaic Device Evaluation

**Figure 7:**
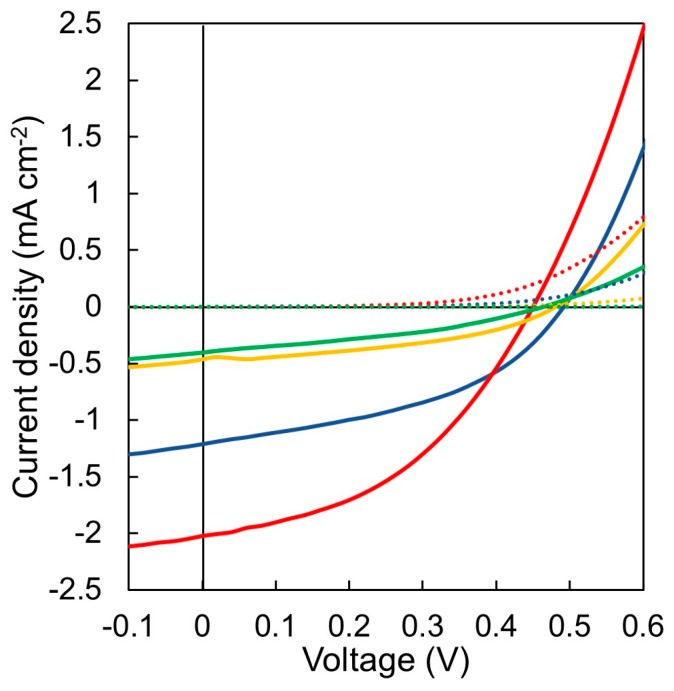
*J*-*V* characteristics of P3HT/P(NDI2OD-T2) (blue), P3HT-*b*-PSt/P(NDI2OD-T2)-*b*-PSt (red), P3HT-*b*-PSt/P(NDI2OD-T2) (yellow) and P3HT/P(NDI2OD-T2)-*b*-PSt (green) exhibiting the best power-conversion efficiency (PCE) of each. Solid and dotted lines represent light and dark conditions, respectively.

In order to confirm the effect of introducing PSt block, the OPV devices with four types of active layers were examined; P3HT/PSt, P3HT-*b*-PSt/P(NDI2OD-T2)-*b*-PSt, P3HT-*b*-PSt/P(NDI2OD-T2), P3HT/P(NDI2OD-T2)-*b*-PSt. The solar cell performances of the devices are summarized in Table 2. Figure 7 shows the current density-voltage (*J*-*V*) curves of the devices showing the best PCE for each. The value of *J*_SC_ increased from 1.16 to 1.73 mA cm^−2^ by introducing the PSt block, while *V*_OC_ and fill factor (FF) were not significantly changed. The reason for increased *J*_SC_ is considered to be that the charge separation efficiency was improved because the heterojunction interface between p- and n-type polymers increased due to the decrease in the domain size of P3HT and P(NDI2OD-T2) segment as explained above. It was also found from SCLC measurements that charge mobility of both p- and n-type polymers is improved by introducing a block of PSt. It is considered that one of the reasons for the improvement of the *J*_SC_ is that the improved charge mobility decreased the probability for the recombination of the separated charges. Hence, the best PCE of 0.39% was attained for the device with active layer of P3HT-*b*-PSt and P(NDI2OD-T2)-*b*-PSt blended. Furthermore, the values of *J*_SC_ and FF decreased from 1.16 to 0.32 mA cm^−2^ and from 0.41 to 0.36, respectively, by introducing PSt only into P3HT. Similarly, the value of *J*_SC_ decreased from 1.16 to 0.25 mA cm^−2^ and the value of FF decreased from 0.41 to 0.35 by introducing PSt only into P(NDI2OD-T2). The reason for these *J*_SC_ decreases is that the presence of PSt block does not reduce the domain size in these cases, and the PSt domain just plays a role as an insulator. Assuming that in both cases, the domains with PSt segment resulted from phase separation in a large scale are covered with PSt domain possessing highly solvating power because of the exclusion of the solvent (chlorobenzene) during the crystallization from the solution, segregated PSt segments at the interface between p- and n-type polymers inhibits the charge separation. It is also speculated that the decrease of FF in these systems is due to the disruption of interpenetrating structure resulting from the formation of above mentioned macroscopic domains leading to the lack of appropriate charge transporting path. As a result, the devices based on the blends, P3HT-*b*-PSt/P(NDI2OD-T2) and P3HT/P(NDI2OD-T2)-*b*-PSt showed lower PCE than P3HT/P(NDI2OD-T2).

The effect of chain length on the OPV performance was investigated by the evaluation of the device comprising P3HT-l/P(NDI2OD-T2)-l or P3HT-l-*b*-PSt/P(NDI2OD-T2)-l-*b*-PSt. The former device exhibited the slightly lower performance than that of P3HT/P(NDI2OD-T2) because of the low molecular weight nature. Performance of the latter device, however, drastically became worse compared with the P3HT-*b*-PSt/P(NDI2OD-T2)-*b*-PSt-based device. In XRD profile for P3HT-l-*b*-PSt/P(NDI2OD-T2)-l-*b*-PSt blend, (100) diffraction derived from P(NDI2OD-T2) segment disappeared (see Figure S4). P(NDI2OD-T2)-l-*b*-PSt is a triblock copolymer, and it is reported that crystallinity more extensively decreased with the increase of polyisoprene (PI) content in PI-*b*-P3HT-*b*-PI triblock copolymers compared with corresponding diblock copolymers [33]. In addition, the intensity of (100) diffraction from the P3HT segment decreased. The reduced crystallinity decreases charge transporting properties, and higher content of insulating PSt component decreases the probability of charge separation. It is considered that both factors made the OPV performance worse.

## 4. Conclusions

An n-type of block copolymer, P(NDI2OD-T2)-*b*-PSt was successfully synthesized and characterized. Electron mobility enhanced by a factor of 8 compared with homopolymer precursor. OPV performance was evaluated for the devices based on the blends P3HT/P(NDI2OD-T2), P3HT-*b*-PSt/P(NDI2OD-T2)-*b*-PSt, P3HT/P(NDI2OD-T2)-*b*-PSt, and P3HT-*b*-PSt/P(NDI2OD-T2) from *J*-*V* characteristics, it was confirmed that the introduction of PSt into both P3HT and P(NDI2OD-T2) improves *J*_SC_ and PCE. From AFM images, it is clarified that the value of RMS decreases in the film of block copolymers blend compared with homopolymers blend resulting from the improvement of the uniformity. It is considered that the probability of charge separation increased despite the presence of the PSt domain at the interface, and the recombination of carriers was suppressed by the enhanced charge mobilities in both domains. On the other hand, when the PSt block was incorporated into only one of the homopolymers, lower PCEs are observed compared with that based on homopolymer blends. This is probably due to the decrease of charge separation resulting from PSt segregation at the interface between p- and n- domains with a large scale, and the disruption of interpenetrated structure leading to the lack of appropriate charge transporting path. Block copolymers containing shorter semiconducting moiety, and higher content of PSt afforded the device with poorer performance due to the reduced crystallinity in both P3HT and P(NDI2OD-T2) domains, and insulating nature of PSt with higher content. 

## Figures and Tables

**Table 1:** Characteristics of P(NDI2OD-T2) and P(NDI2OD-T2)-*b*-PSt.

Polymer	*λ*_max_ (nm) ^1^	LUMO (eV) ^2^	SCLC Electron Mobility (cm^2^ V^−1^ s^−1^) ^3^
P(NDI2OD-T2)	369, 383, 610	−4.0	4.5 × 10^−^^6^
P(NDI2OD-T2)-*b*-PSt	369, 384, 618	−4.1	3.6 × 10^−^^5^

^1^ Wavelength at absorption maxima in chlorobenzene solution (0.01 mg/mL), ^2^ energy level of lowest unoccupied molecular orbital, ^3^ average values determined for 4 devices.

**Table 2:** Photovoltaic parameters of the devices.

Polymers	*V*_OC_^1^ (V)	*J*_SC_^1^ (mA cm^−2^)	FF ^1^	PCE (%)	
				Best	Average
P3HT/P(NDI2OD-T2)	0.50 ± 0.004	1.16 ± 0.05	0.42 ± 0.02	0.26	0.24 ± 0.02
P3HT-l/P(NDI2OD-T2)-l	0.53 ± 0.008	1.07 ± 0.06	0.35 ± 0.02	0.21	0.20 ± 0.02
P3HT-*b*-PSt/P(NDI2OD-T2)-*b*-PSt	0.45 ± 0.005	1.73 ± 0.20	0.41 ± 0.02	0.39	0.32 ± 0.07
P3HT-l-*b*-PSt/P(NDI2OD-T2)-l-*b*-PSt	0.55 ± 0.005	0.78 ± 0.06	0.31 ± 0.04	0.16	0.13 ± 0.03
P3HT-*b*-PSt/P(NDI2OD-T2)	0.47 ± 0.007	0.32 ± 0.13	0.36 ± 0.05	0.10	0.06 ± 0.04
P3HT/P(NDI2OD-T2)-*b*-PSt	0.46 ± 0.006	0.25 ± 0.12	0.35 ± 0.02	0.07	0.04 ± 0.03

^1^ The average values for 4 or 8 devices.

## Supplementary Materials

The following are available online at http://www.mdpi.com/1996-1944/11/3/343/s1, Figure S1: ^1^H NMR spectrum of P(NDI2OD-T2) in CDCl_3_ at 500 MHz, Figure S2: ^1^H NMR spectrum of P(NDI2OD-T2)-l-*b*-PSt in CDCl_3_ at 500 MHz, Figure S3: Cyclic voltammograms of P(NDI2OD-T2) (gray) and P(NDI2OD-T2)-*b*-PSt (orange) (forth scan in each), Figure S4: X-ray diffraction profiles for P3HT-*b*-PSt/P(NDI2OD-T2)-*b*-PSt (red) and P3HT-l-*b*-PSt/P(NDI2OD-T2)-l-*b*-PSt (dark gray) thin films in out-of-plane geometry.
