# Supplementary material for: Fabrication of Completely Polymer-Based Solar Cells with p- and n-Type Semiconducting Block Copolymers with Electrically Inert Polystyrene
Source: Materials (Basel). 2018 Feb 27;11(3):343. doi: 10.3390/ma11030343 (PMC5872922; doi:10.3390/ma11030343)
Supplement: Supplementary file 1 [file materials-11-00343-s001.pdf]

Supplementary Materials

Fabrication of Completely Polymer-Based Solar Cells with p- and n-Type Semiconducting Block Copolymers with Electrically Inert Polystyrene

Eri Tomita, Shinji Kanehashi, and Kenji Ogino*

Graduate School of Bio-Applications and Systems Engineering,

Tokyo University of Agriculture and Technology,

2-24-16 Nakacho Koganei, Tokyo 184-8588, Japan

* Correspondence: kogino@cc.tuat.ac.jp; Tel/Fax: +81-42-388-7404

(1) ^{1}H NMR of P(NDI2OD-T2)

^{1}H NMR ($CDCl_3$ 500 MHz): δ: 8.43-8.86 (m, br, 2H), 7.16-7.45 (br, 4H), 4.11 (br, 2H), 2.00 (br, 2H), 1.16-1.48 (br, 64H), 0.84 (br, 12H). GPC: M_n = 10,100 g mol^{-1}, PDI = 3.33.

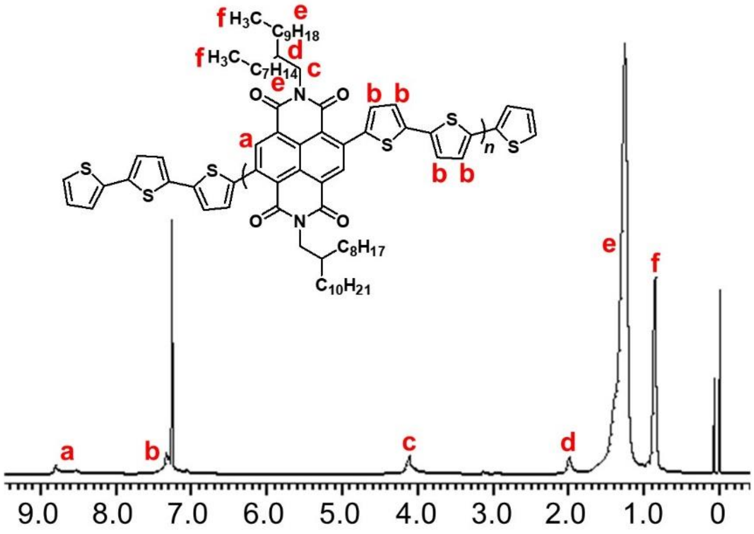

Figure S1. ^{1}H NMR spectrum of P(NDI2OD-T2) in $CDCl_3$ at 500 MHz.

(2) Synthesis of P(NDI2OD-T2)-L-*b*-PSt

Low molecular weight of homopolymer, P(NDI2OD-T2)-L, was also obtained by collecting the hexane soluble fraction in Soxhlet extraction. Extracted hexane solution was added slowly to methanol. The precipitates were collected by filtration, and dried in

vacuum, leading to a deep blue solid as the product (yield 44.2%). ^{1}H NMR ($CDCl_3$ 500 MHz): δ: 8.43-8.86 (m, br, 2H), 7.16-7.45 (br, 4H), 4.11 (br, 2H), 2.00 (br, 2H), 1.16-1.48 (br, 64H), 0.84 (br, 12H). GPC: M_n = 3,880 g mol^{-1}, PDI = 1.94.

Bromination and Suzuki-Miyaura coupling reaction of P(NDI2OD-T2)-L with boronic ester terminated PSt (M_n ; 620 g mol^{-1}, PDI; 1.94) were carried out as described in Subsection 2.1 in the main text. P(NDI2OD-T2)-L-Br_2 (120 mg), boronic ester terminated PSt (176 mg), $Pd(PPh_3)_4$ (13 mg, 0.011 mmol), 2.5 M K_2CO_3 (2.1 mL), toluene (6.3 mL) were placed into a flask equipped with a condenser and a nitrogen inlet, followed by freeze-and-thaw cycles to eliminate air in the mixture. Then the mixture was stirred for 3 days at 90 °C under nitrogen atmosphere. The reaction mixture was precipitated in methanol to recover the product. Reprecipitations in methanol and hot acetone were successively carried out twice each. Then, Soxhlet extraction was carried out using in the order of methanol, acetone and chloroform, and a solution of chloroform was poured into methanol to recover the final product. Yield: 82 mg (57.8%). The weight ratio of PSt segments in P(NDI2OD-T2)-L-*b*-PSt was 26.9 %.

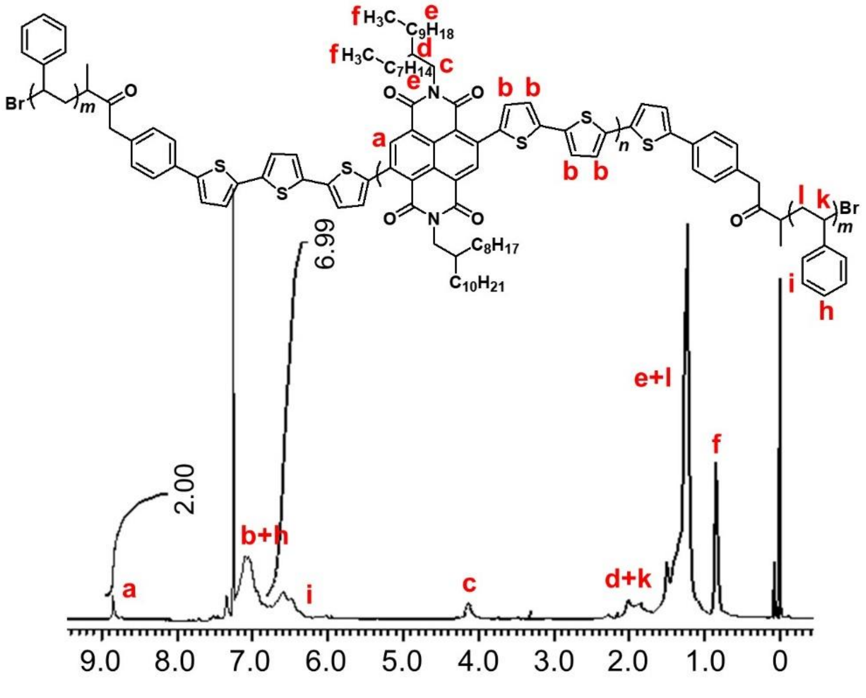

Figure S2. ^{1}H NMR spectrum of P(NDI2OD-T2)-L-*b*-PSt in $CDCl_3$ at 500 MHz.

(3) Calculation of LUMO level

LUMO levels of P(NDI2OD-T2) precursor and P(NDI2OD-T2)-*b*-PSt was determined from by the cyclic voltammograms (CV) (**Figure S3**) using the value of the

onset of the reduction wave (V_{red}) utilizing equation S1 [1]

$$\text{LUMO (eV)} = -(V_{red}+V_{(Ag/AgCl\ vs\ SHE)}+V_{(SHE\ vs\ vacuum)})$$
$$= -(V_{red}+0.198+4.45) \qquad (S1)$$

where V_{red}, $V_{(Ag/AgCl\ vs\ SHE)}$, and $V_{(SHE\ vs\ vacuum)}$ are the potentials of the substrate reduction, Ag/AgCl reference electrode, and standard hydrogen electrode, respectively.

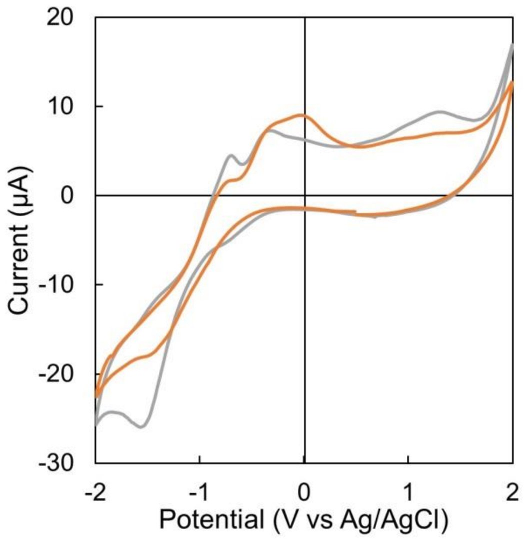

Figure S3. Cyclic voltammograms of P(NDI2OD-T2) (gray) and P(NDI2OD-T2)-*b*-PSt (orange) (forth scan in each).

(4) Molecular weight effect of P(NDI2OD-T2) segment on GIWAXD profiles

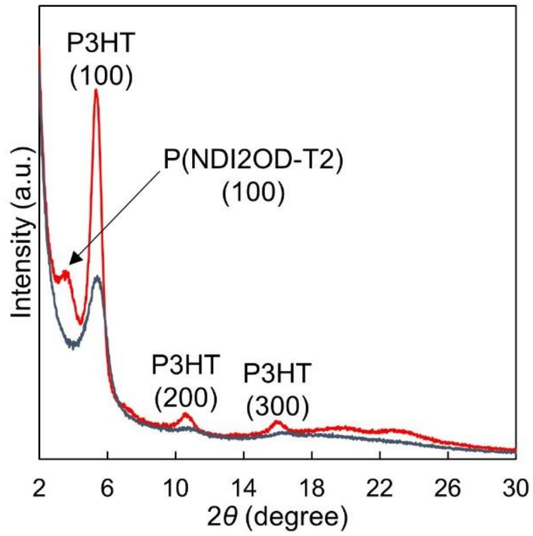

Figure S4. X-ray diffraction profiles for P3HT-*b*-PSt/P(NDI2OD-T2)-*b*-PSt (red) and P3HT-L-*b*-PSt/P(NDI2OD-T2)-L-*b*-PSt (dark gray) thin films in out-of-plane geometry.

References

[1] Kim, K.; Ohata, R.; Kanehashi, S.; Tsuchiya, K.; Ogino, K. Hole transporting properties of cyclic pentamer of 4-butyltriphenylamine. *Chem. Lett*. **2017**, *46*, 1154-1156, 10.1246/cl.170412
